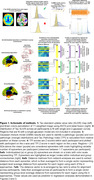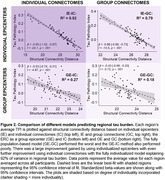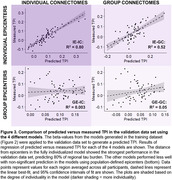# Support for transmission of tau pathology in humans using individualized epicenters and structural connectomes

**DOI:** 10.1002/alz.094052

**Published:** 2025-01-09

**Authors:** Christopher Brown, Sandhitsu R. Das, Ilya M. Nasrallah, John A. Detre, Paul A. Yushkevich, Corey T McMillan, David A Wolk

**Affiliations:** ^1^ University of Pennsylvania, Philadelphia, PA USA; ^2^ Penn Image Computing and Science Laboratory (PICSL), University of Pennsylvania, Philadelphia, PA USA; ^3^ Department of Radiology, University of Pennsylvania, Philadelphia, PA USA; ^4^ Department of Neurology, University of Pennsylvania, Philadelphia, PA USA; ^5^ Perelman School of Medicine, University of Pennsylvania, Philadelphia, PA USA

## Abstract

**Background:**

Population‐based functional connectomes help explain heterogeneity in tau spread in Alzheimer’s disease by demonstrating spread among connected neurons from canonical epicenters. However, if the hypothesis of cell‐to‐cell transmission of tau is correct, individual structural connectomes seeded from individual‐specific epicenters of PET‐tau pathology should improve prediction of regional tau patterns.

**Method:**

86 participants from the Penn Aging Brain Cohort and 158 participants from ADNI with multi‐shell diffusion MRI and AV1451 Tau PET within 18 months of each other were included. A schematic of methods is shown in Figure 1. Tau PET standard uptake value ratios were converted to Tau Pathology Indices (TPI) based on Gaussian‐mixed models. Regions > 2.5 standard deviations above the mean TPI within an individual were used as individualized epicenters, while entorhinal cortex (ERC) was used as a canonical group epicenter. Probabilistic tractography generated individual connectomes and a group connectome in amyloid‐negative participants. Network‐analysis calculated structural connectivity distances from epicenters for each region. Data were re‐arranged by tau pathology rank to average across all participants using each combination of individualized and group epicenters and connectomes. The model of Regional TPI = ß*Distance + e was tested in the training dataset using each of the 4 approaches: 1) Group canonical epicenter and group connectome (GE‐GC), 2) Group canonical epicenter and individual connectome (GE‐IC), 3) Individualized epicenters and group connectome (IE‐GC), and 4) Individualized epicenters and connectomes (IE‐IC). The resulting models were then applied to the validation dataset to predict regional TPI and compare to actual TPI.

**Result:**

The fully individualized model demonstrated a significantly stronger relationship between connectivity and regional tau burden (ß = ‐0.95[‐1.03, ‐0.87]) compared to all other models (Z = ‐5.81, p < .001, Figure 2). When the four trained models were applied to the validation dataset, the fully individualized model explained 80% of regional TPI variance, while the other models explained = 52% (Figure 3).

**Conclusion:**

Individualized structural connectomes and tau epicenters capture the heterogeneity of Alzheimer’s disease and provide strong support for the cell‐to‐cell transmission of tau pathology in vivo.